# How reproducible are clinical measurements in robotic knee surgery?

**DOI:** 10.1186/s40634-023-00582-3

**Published:** 2023-03-24

**Authors:** Fabrizio Matassi, Edoardo Bori, Niccolò Giabbani, Roberto Civinini, Bernardo Innocenti

**Affiliations:** 1 Orthopedic Clinic, University of Florence, AOU Careggi, Florence, Italy; 2 BEAMS Department (Bio Electro and Mechanical Systems), École Polytechnique de Bruxelles, Université Libre de Bruxelles, Bruxelles, Belgium

**Keywords:** Robotic surgery, Knee surgery, Reproducibility, Variability, In vivo measurements

## Abstract

**Purpose:**

Robotic-assisted surgery has been recently introduced to improve biomechanical restoration, and thus better clinical and functional outcomes, after knee joint arthroplasty operations. Robotic-assisted uni-compartmental knee arthroplasty (UKA) aims indeed to improve surgical bone resection and alignment accuracy, optimized component positioning and knee balancing, relying on a series of calibration measurements performed during the surgery. These advantages focus therefore on improving the reproducibility of UKA surgeries, reducing (if not eliminating) eventual differences among high- and low-volume surgeons.

The purpose of this study is to investigate and quantify the reproducibility of in-vivo measurements performed with a robotic system: the intra- and inter-observer variability of a series of measurements was therefore analyzed and compared among differently experienced operators.

**Methods:**

Five patients were analyzed and underwent robotic-assisted UKA using a semi-active robotic system.

Three different observers with different experience levels were involved to independently perform the measurements of two parameters of the preoperative knee (Hip-Knee-Ankle angle [HKAa], Internal-External Rotation) at different degrees of knee flexion. Inter-observer and intra-observer comparisons were performed.

**Results:**

The average variability in the measurements obtained from the intra-observer and inter-observer comparisons were always < 0.68° for HKAa and < 2.59° for internal-external rotation, and the ICCs showed excellent agreement (> 0.75) for most cases and good agreement (> 0.60) in the remaining ones.

**Conclusion:**

This study demonstrated high reproducibility of the measurements obtainable in clinical environment with the robotic system. The inter-observer results furthermore showed that the level of confidence with the robotic system is not significantly influencing the measurement.

## Introduction

Over recent years, unicompartmental knee arthroplasty (UKA) has gained an increasing popularity due to the encouraging results reported in literature and national arthroplasty registries [17]. UKA accounts for 10% of all cases of knee arthroplasty worldwide and is expected to increase to more than 20% in the future [22].

Medial unicompartmental knee arthroplasty, in particular, represents a suitable treatment for patients with isolated end-stage medial knee arthritis or osteonecrosis of the medial compartments of the knee and it is able to provide durable pain relief and functional improvement in more than 90% of patients [13, 16].

Nevertheless, the functional outcomes and survival rate of UKA are strictly depending on many factors such as patient selection, prosthetic design, polyethylene quality and, moreover, implant alignment and fixation [16]. Indeed, limb overall malalignment and tibial and femoral components malposition is poorly tolerated in UKA and can jeopardize long-term survival: these factors contribute to alter drastically the stress distributions in the knee, influencing the progression of osteoarthritis in the opposite compartment of the joint and increasing wear of the polyethylene insert, thus leading to the need of a revision surgery [3, 8–10].

Robotic-assisted surgery has been recently introduced with the aim of improving the results of joint arthroplasty, helping the surgeons in restoring patients’ functional kinematics [17, 22]. Robotic surgery has reached a widespread application and interest, highlighted by the increasing number of annual publications on this topic (from 2500 to 6500 in the last 5 years) [11].

Robotic-assisted UKA aims to provide enhanced surgical bone resection and alignment accuracy, optimized component positioning and knee balancing, with the goal of enhancing patient clinical and functional outcomes [3, 9, 11, 17]. These advantages focus therefore on improving the reproducibility of UKA surgeries, reducing (if not eliminating) eventual differences among high- and low-volume surgeons [18].

Some robotic systems, in detail, require the surgeon to record anatomical landmarks during the actual procedure to match them with the preoperative CT-scan and guide the surgeon during relative planned bone resections; the bone cuts are consequently performed and the prosthetic components positioned accordingly to preplanning, with claimed higher precision respect to conventional UKA surgeries [1, 7, 23]. In order to achieve the sought positioning, therefore, the intraoperative measurement of the knee landmarks and clinically relevant parameters represents one of the most important stages in the surgical room as on this information are based all the further operation steps. The advantages of the robotic surgery in terms of component’s positioning and knee alignment have been recently documented and reported in literature [1, 17, 20] but the reproducibility of the robotic measurement procedures to obtain the knee parameters in the surgical room has not been addressed yet. As positioning of the implant and balancing of the knee depend intrinsically on these measurements, their consistency and subsequent functional and clinical outcomes depend on the robotic measurements’ reproducibility too.

Aiming to provide a reference for the surgeons interested in performing operations with robotic assistance (and also to surgeons already performing them), the purpose of the current study is to investigate and quantify the reproducibility of the in vivo measurements performed intra-operatively with a robotic system: in order to achieve this goal, the intra- and inter-observer variability of a series of measurements obtained with a robotic system was therefore analyzed and compared among differently experienced operators.

## Materials and methods

### Study design

Between September 2021 and November 2021, five consecutive patients underwent robotic-assisted UKA at our Institution, using a semi-active robotic system (MAKO software, MAKO Surgical Corp, Stryker, Kalamazoo, Michigan, USA) and MCK Restoris Knee implant (Stryker, Kalamazoo, Michigan, USA). The robotic system uses optical motion capture technology to dynamically track marker arrays (that are fixed to the femur and the tibia) and to provide dynamic referencing for the femur and tibia three-dimensional haptic bone resection [3].

The indication for surgery was symptomatic isolated medial osteoarthritis (Kellgren-Lawrence grade III–IV) [14], reducible deformity, stability in sagittal and coronal plane, no previous knee surgery. Contraindications were lateral or patellar osteoarthritis, inflammatory arthritis, ligament insufficiency or fixed flexion or varus deformity > 10 degrees. Two out of five patients were male, the average age was 67.20 (standard deviation: 6.98) and the average Body Mass Index (BMI) was 28.10*.*

The number of patients involved in the study was chosen in agreement with other studies on similar topics available in literature [6, 12, 24], all focused on defining the precision of different measurement or reconstruction processes as the one addressed in this study.

All surgical procedures following the measurement procedures were performed at our Institution by high-volume senior surgeon with experience with robotic-assisted UKA. The study and follow-up, respecting the criteria of the Declaration of Helsinki, have been approved by Institutional Review Board (IRB) of Azienda Ospedaliera Universitaria Careggi (AOUC) Department of Surgery and Translational Medicine and all selected patients were properly informed before surgery about the treatment and follow-up visits after discharge.

### Measurements

Two parameters of the knee were recorded in real time by the system software preoperatively (thus before the actual bone cuts and osteophytes removal) and stored for subsequent analysis:

- the Hip-Knee-Ankle angle (HKAa);
- the Internal-External Rotation of the joint.

The HKA angle is the angle between the mechanical axis of femur and the mechanical axis of tibia, and conventionally can be expressed as its angular deviation from 180° (neutral mechanical axis); it is measured from the software by relying on the positions of the center of hip, center of the knee and center of ankle joints which are previously defined during the robotic system calibration [3].

The internal-external rotation of the joint is instead measured as rotation in the axial plane between the femoral and the tibial medio-lateral axes [24]; external rotation was assigned a positive value, while internal rotation was assigned a negative one.

The two parameters addressed were collected at 0°, 30°, 60°, 90° and 120° degrees of flexion; the choice of the analyzed angles was made in order to address full extension, mid-flexion (45°/60°), 90° of flexion and deep-flexion, configurations that reflect the behavior of the knee throughout its entire range of motion.

All measurements were independently done by three different observers, defined according to their level of confidence with the robotic system [3]:

- observer 1: high-volume surgeon (performing more than 30 surgeries per year);
- observer 2: low-volume surgeon (performing among 5 and 30 surgeries per year);
- observer 3: orthopedic resident (performing less than 5 surgeries per year).

This choice was made with the aim of being able to quantify the influence of the observer’s experience on the results of the procedure. All the measurements were performed following the same routine protocol: a hand was positioned below the popliteal area while the other hand securely grabbed the foot-holder, with the foot kept in neutral position and no varus or valgus stress practiced; the robotic system then recorded the sought information and prompted on screen the following flexion angle required. It is important that the foot is neutrally rotated (not stressed internally or externally) as the joint is flexed, since these rotations could lead to inaccurate readings.

Starting from complete extension (0°), the knee was then progressively flexed to collect the data at each other required angle.

Each observer collected the series of measurements five times, restarting from 0° of flexion once the previous series was completed. The measurements were performed in order of increasing experience of the observer (i.e. Observer 3, Observer 2 and lastly Observer 1, whose measurements were then used for the actual surgical operation), in order to avoid any influence of the more experienced surgeon on the less experienced one measurements performances.

The resulting data were analyzed to extract intra- and inter-observer variability.

### Surgical steps

The surgical technique followed for the cases analyzed is described in detail in [11, 17].

All patients received a preoperative CT of the affected knee to build a 3D CAD-model of the patient’s knee. Surgical planning was performed preoperatively by the senior surgeon and the engineer using the robotic software, which allows the identification of the components positioning and size required to achieve optimal knee alignment and balancing in flexion and extension.

The UKA operation was then performed following the medial mini-parapatellar [19]. Femoral and tibial registration pins were inserted, and the required set of anatomical landmarks was recorded and matched with the CT model; the measurements of the native knee coronal alignment and rotation were then made via the system software and registered (the process is described in detail in the relative chapter). After having chosen and fine-tuned the desired alignment, rotation, and ligament balancing, the planned bone resections were performed with the robotic arm assistance and the prosthetic components were positioned. At the end of the whole procedure, the afore mentioned parameters were measured again following the same protocol.

### Statistical analysis

Normality of distribution was assessed by applying the Shapiro–Wilk normality test. All data met the normality requirements for parametric statistics and were thus summarized as mean ± standard deviation.

To test the reproducibility and repeatability of the proposed technique, the intra- and inter-class correlation coefficients (ICCs) were calculated together with their respective Confidence Interval for the HKA and for the Internal-External Rotation [5]. The ICC (calculated using k-Rating, Absolute-Agreement, 2-Way Mixed-Effects Model) is by definition measured in a range from 0 to 1, with a value close to 1 indicating better agreement: an ICC > 0.75 indicates excellent agreement, while an ICC > 0.60 is taken as a threshold for a good agreement [15]. All the statistical analysis were performed using MatLab R2021b (The MathWorks, Natick, Massachusetts, USA).

## Results

The variability and ICCs resulting from the HKAa and the internal-external rotations measurements are reported in Tables 1, 2, 3 and 4 in terms of average, standard deviation (SD) and maximum, both for intra-observer and inter-observer.

**Table 1:**
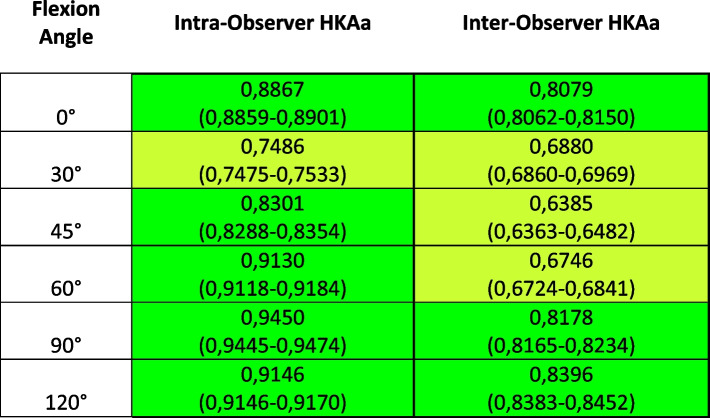
ICC results for HKAa measurements and relative Confidence Interval; highlighted in green, the ICC representing excellent agreement (> 0.75), in light green the ones representing good agreement (> 0.60)

**Table 2:**
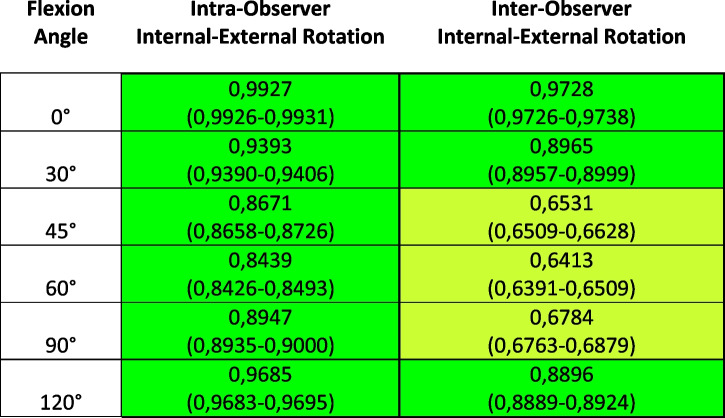
ICC results for Internal-External Rotation measurements and relative Confidence Interval; highlighted in green, the ICC representing excellent agreement (> 0.75), in light green the ones representing good agreement (> 0.60)

**Table 3:** Intra-observer and inter-observer variability for HKAa measurements

Flexion Angle	Intra-Observer			Inter-Observer		
	Average [°]	SD [°]	Max [°]	Average [°]	SD [°]	Max [°]
0°	0,37	0,45	2,00	0,36	0,50	2,53
30°	0,51	0,48	2,30	0,55	0,61	3,07
45°	0,40	0,49	2,10	0,68	0,71	3,60
60°	0,30	0,27	1,10	0,60	0,54	2,17
90°	0,24	0,20	0,90	0,44	0,34	1,27
120°	0,28	0,26	1,60	0,44	0,29	1,23

**Table 4:** Intra-observer and inter-observer variability for internal-external rotation measurements

Flexion Angle	Intra-Observer			Inter-Observer		
	Average [°]	SD [°]	Max [°]	Average [°]	SD [°]	Max [°]
0°	0,43	0,40	1,90	0,82	0,61	3,03
30°	1,02	0,86	3,20	1,32	1,15	5,43
45°	1,03	0,94	3,60	1,83	1,47	5,60
60°	1,35	1,15	4,40	2,35	1,58	7,47
90°	1,38	1,05	4,20	2,59	1,71	6,43
120°	1,11	0,76	3,10	2,01	1,35	5,37

Addressing the intraoperative measurement of HKAa, the results showed highly reproducibility both for the same surgeon (intra-observer measurements average variability < 1° for all flexion angles), both among different surgeons with different level of robotic-surgery experience (inter-observer measurements average variability < 1° for all flexion angles) [15].

Analyzing the different flexion angles in detail, the intra-observer ICC returned to be higher than 0,75 (and thus falling in the “excellent agreement”) for all angles, with the exception of measurements at 30° (0,74); the inter-observer variability was instead lower than 0,75 only for measurements from 30° to 60° of flexion, however still being > 0.60 and therefore in the “good agreement” class [15].

Addressing instead the measurements of internal-external rotation, instead, it resulted that the intra-observer mean variability was less than 1,5° for all flexion angles, with the lowest values found for the measurements performed in full-extension (0,43°); the inter-observer mean variability returned to be slightly higher, but always lower than 2,5° regardless of the flexion angle.

## Discussion

The result obtained in terms of HKAa showed higher repeatability for the measurements at the flexion angles typically taken as reference during the calibration (0° and 90°) [21], hence justifying this choice as they are able to provide reliable and robust info on which base the surgical cuts. Flexion angles like 30° and 60°, on the other hand, suffer the effects of the so called mid-flexion instability [21] and therefore are characterized by higher variability if compared to full-extension and 90° configurations, but it is noticeable that also in these sub-optimal cases the comparison did not return remarkable differences.

It can thus be concluded that HKAa measurements are highly reproducible, especially when performed at the flexion angles mostly used as reference in the clinical practice.

The results obtained for internal-external rotation showed instead that these measurements involve a higher level of variability if compared to HKAa, and it is remarkable the fact that this is true also for the intra-observer measurements: this means therefore that the operation may require more experience if compared to the one performed for the HKAa, since even the same observer may incur in incongruities in performing the measurement multiple times.

Even if characterized by a higher variability compared to HKAa, it is to highlight that the ICC for internal-external rotations returned to be excellent as higher than 0,75 for both intra- and inter-observer measurements for almost all flexion angles (with the exclusion of 45°, 60° and 90° for inter-observer); these results confirm therefore the capacity of the robotic system to provide the surgeon the possibility to precisely measure knee rotational status, therefore avoiding excessive rotational malalignment with its potential detrimental effect [2, 4].

The present study has however limitations worthy to be noted: the sample size considered, even if representative of a more numerous population, could indeed can be increased and similar limitation is represented by the fact that only a single brand of robotic systems and the relative brand of prostheses were used.

By analyzing and comparing multiple in-vivo intraoperative measurements of the main knee biomechanical parameters (HKAa and rotation) at different predefined angles of knee flexion, this study demonstrated a considerably high level of reproducibility of this operation both for the same observer and for observers with different levels of surgical-robotic experience.

The inter-observer repeatability is indeed of paramount importance, as it is fundamental to highlight that the “precision of the measurement” is related to the combination “observer-robotic system” and not to the surgical robot alone. The role of the surgeon in indeed more than being a passive observer as it covers an active function in the measuring operation, and therefore the variability in user’s experience becomes a relevant factor to consider when trying to quantify the precision of the measuring tool as a whole.

It is finally important to highlight that these results were obtained during actual surgeries rather than in a laboratory experimental activity (thus performed in a controlled environment); this aspect indeed allows to correlate this study directly to the surgical use of the robotic system, improving further the relevance of the obtained results.

## Conclusions

This study demonstrated the high intra and inter-observer reproducibility of the measurements of knee HKAa and internal-external rotation obtainable in a clinical environment with the use of a robotic system, highlighting the great reliability of the robotic system in acquiring the data then used for the operation itself. Furthermore, the resulting low inter-observer variability provided a significant insight on the level of confidence with the robotic system required to successfully perform the intra-operatively measurement with this technique.
