# Correction: How reproducible are clinical measurements in robotic knee surgery?

**DOI:** 10.1186/s40634-023-00609-9

**Published:** 2023-04-19

**Authors:** Fabrizio Matassi, Edoardo Bori, Niccolò Giabbani, Roberto Civinini, Bernardo Innocenti

**Affiliations:** 1 Orthopedic Clinic, University of Florence, AOU Careggi, Florence, Italy; 2 BEAMS Department (Bio Electro and Mechanical Systems), École Polytechnique de Bruxelles, Université Libre de Bruxelles, Bruxelles, Belgium

**Correction: J EXP ORTOP 10, 32 (2023)**

10.1186/s40634-023-00582-3

Following publication of the original article [1], the authors’ given name and family name were switched.

Matassi Fabrizio, Bori Edoardo, Giabbani Niccolò, Civinini Roberto and Innocenti Bernardo

The correct authors’ name are shown in the authorgroup section of this article and the original article [1] has been corrected.
